# Development of an AI-based model for sex estimation using CT-derived metrics from paranasal sinuses

**DOI:** 10.1007/s00414-026-03789-y

**Published:** 2026-04-06

**Authors:** Diego Santiago de Mendonça, Marcela Lima Gurgel, Esther Carneiro Ribeiro, João Victor de Oliveira Rodrigues, Andrea Silvia Walter de Aguiar, Lucia Helena Soares Cevidanes, Lúcio Mitsuo Kurita, Fabrício Mesquita Tuji, Frederico Sampaio Neves, Paulo Goberlânio de Barros Silva, Saulo Anderson Freitas de Oliveira, José Wellington Franco da Silva, Fábio Wildson Gurgel Costa

**Affiliations:** 1 School of Dentistry, Federal University of Ceará, Rua Monsenhor Furtado, 1273, Rodolfo Teófilo, Fortaleza, Ceará Brazil; 2 School of Information Systems, Federal University of Ceará Campus Crateús, Crateús, Ceará Brazil; 3 Department of Orthodontics, Adams School of Dentistry, University of North Carolina, Chapel Hill, NC USA; 4 School of Dentistry, Federal University of Pará, Belém, Pará Brazil; 5 School of Dentistry, Federal University of Bahia, Salvador, Bahia Brazil; 6 University Center UNICHRISTUS, Fortaleza, Ceará Brazil; 7 Department of Computer Science, Federal Institute of Education, Science and Technology of Ceará, Fortaleza, Ceará Brazil

**Keywords:** Sex estimation, Paranasal sinuses, Multislice computed tomography, Machine learning, Forensic anthropology, Morphometric analysis

## Abstract

Sex estimation plays a critical role in the reconstruction of the biological profile in forensic contexts. The paranasal sinuses, owing to their structural complexity and resistance to postmortem degradation, have emerged as valuable anatomical regions for this purpose. This observational, retrospective study assessed the accuracy of sex estimation in Brazilian adults using linear and volumetric measurements of the frontal, maxillary, and sphenoidal sinuses obtained from multislice computed tomography (MSCT). A total of 220 MSCT scans (113 males, 107 females) from three imaging centers were analyzed. Semiautomatic segmentation was performed using ITK-SNAP and 3D Slicer to extract craniometric features. Statistical analyses and supervised machine learning models, including logistic regression, linear support vector machine (SVM), random forest, and k-nearest neighbors, were employed for classification. Males showed significantly larger anteroposterior, supero-anterior, and volumetric dimensions (*p* < 0.05). The highest classification accuracy was achieved by logistic regression and linear SVM models, both reaching 84%. These models outperformed conventional discriminant analysis. The study supports the forensic utility of CT-derived morphometric parameters of the paranasal sinuses for sex estimation and highlights the potential of machine learning as a robust complementary tool in forensic investigations. Findings also underscore the relevance of broader validation in multiethnic forensic contexts.

**Supplementary Information:**

The online version contains supplementary material available at 10.1007/s00414-026-03789-y.

## Introduction

Sex estimation represents a fundamental step in the reconstruction of the biological profile of unidentified individuals in forensic investigations. In forensic anthropology, personal identification is a highly sensitive process based on the comparison between ante-mortem biological data from a presumed individual and post-mortem information obtained from unknown human remains [1]. Traditionally, several anatomical structures, particularly the pelvis and the skull, have been used for sex estimation [2, 3].

Forensic anthropology plays a central role in this field, characterized by the application of physical anthropology principles to the medico-legal identification of human remains. This interdisciplinary science addresses a wide range of issues and contributes significantly to both criminal investigations and humanitarian or human rights contexts, particularly in the identification of victims of mass disasters [4, 5].

A variety of imaging modalities have been used in forensic osteology for sex estimation, including digital radiography, magnetic resonance imaging, and computed tomography (CT) [4]. Among the available methods, CT is particularly noteworthy for significantly improving the effectiveness of forensic anthropologists and pathologists by facilitating quicker and more precise identification of individuals under examination [6]. Previous studies have demonstrated the particular effectiveness of CT in morphometric analyses of the maxillary sinus (MS), which has been recognized as a reliable and rapid method for human identification [7, 8].

The nasal cavity and paranasal sinuses (PNS) exhibit complex anatomical morphology and significant interindividual variation, making them particularly challenging regions for morphometric assessment. CT has become the standard imaging modality for preoperative evaluation and the study of inflammatory sinonasal diseases. With the advent of three-dimensional reconstruction systems, the use of CT has expanded considerably in recent decades, allowing for more precise delineation of anatomical parameters in the sinonasal region. Moreover, studies have shown high agreement between measurements obtained via CT and those derived from anatomical dissection [9].

In recent years, there has been a significant expansion in the application of artificial intelligence (AI) techniques across various scientific fields, including forensic medicine [10]. Within this framework, Tournois et al. [11] identified specific AI applications adopted by forensic pathologists, offering a comprehensive overview of current practices and highlighting potential advancements in specialized areas of legal medicine. Moreover, recent studies have employed machine learning to directly infer attributes such as age or to estimate measurements traditionally made by human experts, enabling the development of complex and accurate regression models related to patient age and other anatomical variables [12, 13]. As sex estimation has increasingly become the focus of such investigations, important contributions have emerged. Various AI-based models and algorithms have been applied in forensic odontology in diverse imaging modalities such as panoramic radiography and CT [14–18].

The present study aimed to evaluate sex estimation based on linear and volumetric measurements of the frontal (FS), maxillary, and sphenoidal (SS) sinuses obtained from multislice computed tomography (MSCT) scans of Brazilian individuals from the North and Northeast regions. It is noteworthy that, to date, no studies have simultaneously evaluated volume and linear parameters, such as distances between planes and predefined anatomical landmarks, in analyses involving the MS, FS, and SS. The classification of individuals as male or female was conducted through different models advanced AI-based approaches.

## Materials and methods

### Study design

A cross-sectional, retrospective observational study was conducted using MSCT scans obtained from diagnostic imaging centers located in the Brazilian states of Pará, Bahia, and Ceará. The study strictly adhered to the guidelines set forth by the Strengthening the Reporting of Observational Studies in Epidemiology (STROBE) initiative for observational research [19], as well as the Transparent Reporting of a Multivariable Prediction Model for Individual Prognosis or Diagnosis statement for studies involving artificial intelligence. Ethical approval was obtained from the local Research Ethics Committee (protocol number 61257622.0.0000.5054), and the study was conducted in accordance with the principles outlined in the Declaration of Helsinki.

### Participants

The sample consisted of 220 multislice CT scans from male and female individuals, sourced from three diagnostic centers located in the North and Northeast regions of Brazil. Two investigators initially reviewed the institutional imaging databases to identify eligible scans, which had been acquired for various clinical indications, including the assessment of craniomaxillofacial trauma.

Scans were selected based on predefined eligibility criteria. Inclusion criteria were: (a) age between 18 and 49 years; (b) presence of a complete upper posterior dentition; (c) tomographic slice thickness not exceeding 2.0 mm; and (d) a field of view (FOV) greater than 180 mm.

Exclusion criteria included: presence of radiological signs suggestive of fractures; sinonasal pathologies capable of altering the anatomical or radiographic pattern of the PNS; presence of metallic artifacts, such as those from dental implants or osteosynthesis hardware; poor image quality impairing clear evaluation of sinus structures; and absence of any PNS region within the scanned volume.

### Setting

Based on the findings of De Mendonça et al. [20], who identified significant differences in the mean diameter of the MSs between males and females in a Brazilian population sample (4.02 ± 0.41 mm vs. 3.82 ± 0.27 mm, respectively), a sample size of 220 CT scans was estimated to achieve 90% statistical power and 95% confidence in the alternative hypothesis of the present study.

Prior to analysis, all CT exams were coded following a sequence generated through simple random allocation using the “randbetween” function in Microsoft^®^ Excel, version 16.11 (Microsoft^®^, Seattle, WA, USA). This process was performed by an external collaborator who had no involvement in the subsequent stages of the study, ensuring a double-blind design. Accordingly, both the examiner and the statistician remained blinded to the sequence of the evaluated scans.

Data collection was performed by a single trained observer (DSM), a radiologist with expertise in oral and maxillofacial imaging. The assessments were conducted in a dimly lit room, with adjustments to screen brightness and contrast allowed during image review. To prevent visual fatigue, a maximum of two tomographic volumes were analyzed per session, as each evaluation required approximately 45 min to complete, following a protocol similar to that employed by De Mendonça et al. [20].

The observer underwent prior calibration training, conducted by experienced researchers in oral and maxillofacial radiology (FWGC and LMK), to ensure reliability. An initial set of randomly selected CT images was analyzed under blinded conditions, and the same dataset was re-evaluated after a 15-day interval to assess intraobserver agreement.

### Data sources, measurements, and variables

Tomographic images were acquired using two different CT scanner models: (1) Somatom Emotion 6 (Siemens, Forchheim, Germany) and (2) Light Speed VCT (GE Healthcare, Piscataway, NJ, USA). Acquisition parameters followed comparable institutional protocols, with table increment of 1 mm, tube voltage of 130 kVp, tube current ranging from 100 to 200 mA, slice thickness up to 2.0 mm, field of view (FOV) of 180 mm, and a rotation time of 0.6 s. To reduce scanner-related variability, image normalization was performed through standardization of the maximum voxel size prior to segmentation, ensuring comparability of linear and volumetric measurements across devices.

**Fig. 1:**
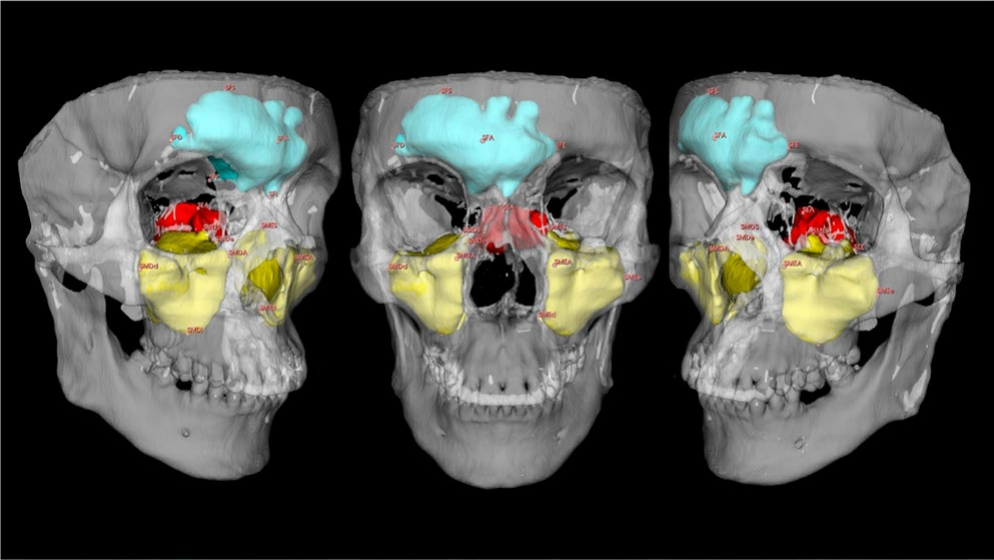
Note. This data is mandatory. Please provide

**Fig. 2:**
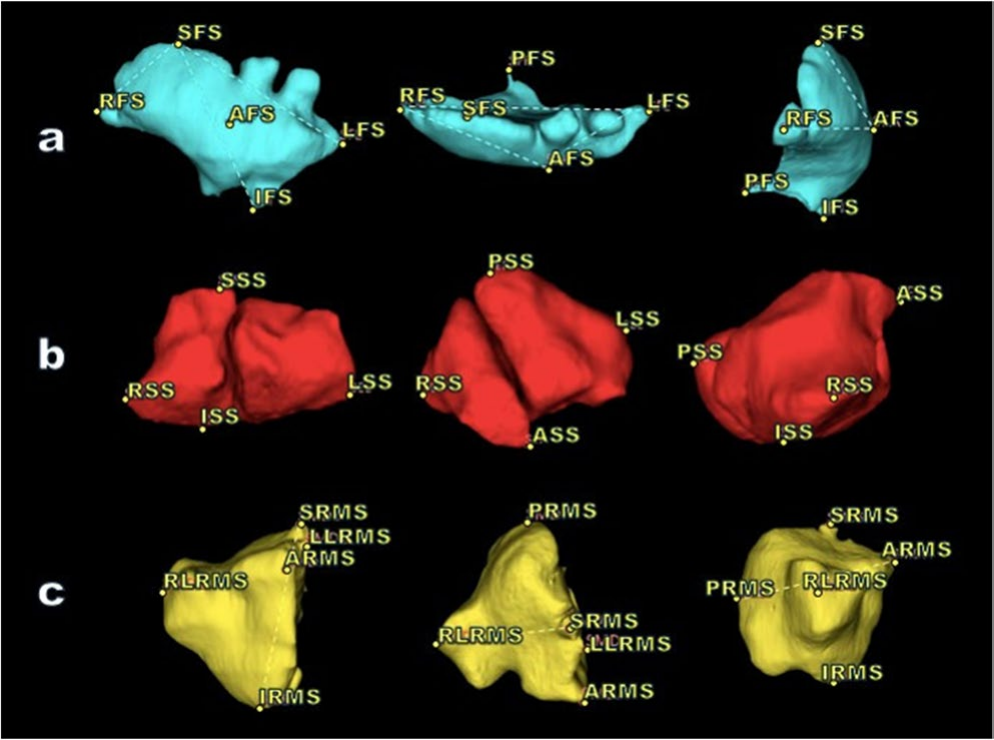
Note. This data is mandatory. Please provide

Both linear and volumetric measurements were obtained and expressed in absolute values (millimeters). In this study, linear measurements were derived using two complementary approaches: (1) projected distances obtained on multiplanar reconstructions (axial, coronal, or sagittal planes) defined by preselected anatomical reference points; and (2) direct three-dimensional distances measured between anatomical landmarks in space. For clarity, linear measurements refer to projected distances between anatomical planes, whereas three-dimensional measurements refer to direct point-to-point Euclidean distances calculated in three-dimensional space. The specific measurements assessed in this study are detailed in Supplementary material 1, and Figs. 1 and 2.

All analyses related to anatomical variations and measurements were performed using a dedicated workstation (Dell Inc., model G3 3590, Intel^®^ Core™ i5-9300 H CPU @ 2.40 GHz, 2400 MHz, 4 cores, 8 logical processors, and HD LED-backlit display), employing the open-source software ITK-SNAP (version 3.8.0) [22] and 3D Slicer (version 4.10.2; www.slicer.org).

The initial step involved orienting and aligning the head within the tomographic images to ensure that all measurements were performed orthogonally to the horizontal plane. Thereafter, axial, sagittal, and coronal slices were reconstructed to support the observer during the evaluation process. A standardized axial reference plane, parallel to the hard palate, identified in the sagittal view and aligned with the anterior and posterior nasal spines, was employed in accordance with the methodology described by Ruellas et al. [21].

Segmentation of the paranasal sinuses was performed using a semiautomatic approach implemented in ITK-SNAP software, version 3.8.0 [22] and 3D Slicer software, version 4.10.2. The files were initially imported into 3D Slicer, in order to standardize patient head positioning. Subsequently, the datasets were converted into the .gipl format in two steps using ITK-SNAP software, version 3.8.0 [22]. Intensity-based thresholding was applied to identify air-filled regions, followed by region-growing tools to delineate the sinus cavities. In ITK-SNAP, active contour (snake) evolution was used to refine segmentation boundaries. All segmentations were visually inspected and manually adjusted, when necessary, to ensure anatomical accuracy. Finally, the volumes of the MS, FS, and SS were obtained directly within ITK-SNAP (Cognitica, Philadelphia, PA, USA), following the methodology previously described by Farias-Gomes et al. [23].

### Bias

To minimize potential sources of bias, a priori sample size calculation was performed to ensure a representative sample with balanced distribution between male and female individuals, thereby reducing the risk of selection bias. Additionally, participant selection was standardized with regard to dental status, and tomographic scans presenting signs suggestive of pathological alterations and/or trauma were excluded, as these factors could act as confounding variables. To further reduce measurement error, all tomographic evaluations were performed by a single, previously calibrated examiner who remained blinded to the sex of the individuals. Furthermore, a reliability assessment of the measurements was conducted to ensure the quality and accuracy of the data obtained.

### Statistical methods

Statistical analyses were performed by a single investigator (PGBS) using SPSS software version 20.0 (IBM Corporation, Armonk, NY, USA), adopting a 95% confidence level. To assess systematic error, the paired t-test, Pearson correlation coefficient, and intraclass correlation coefficient (ICC) were applied. The ICC was calculated using a two-way random-effects model with 95% confidence intervals, and results with p-values < 0.05 were considered statistically significant. Intraobserver reproducibility was classified according to the following thresholds: poor (< 0.5), moderate (0.5–0.74), good (0.75–0.9), and excellent (> 0.9).

Descriptive statistics were calculated for all analyzed parameters, and measurements were compared between sexes. In addition to traditional discriminant analysis, this study employed various machine learning algorithms for sex estimation.

### Machine learning

The application of machine learning techniques to the evaluation of PNS measurements derived from CT was structured into four main stages: preprocessing, model implementation, result evaluation, and refinement.

Initially, image preprocessing involved several procedures aimed at optimizing the dataset for analysis, including data formatting and structuring. When clarification was required regarding specific variables, one of the authors (JVOR) consulted the observer (DSM), who provided the necessary explanations. No outliers were removed, no variables were excluded, and dimensionality reduction techniques were not applied, as the number of input features was limited and manually selected based on anatomical relevance, thereby reducing the risk of overfitting.

Following preprocessing, model implementation was carried out using four supervised machine learning algorithms: Random Forest (RF), Logistic Regression (LR), Support Vector Machine (SVM), and K-Nearest Neighbors (KNN). The models were trained on a labeled dataset incorporating linear, tridimensional, and volumetric measurements of the PNS. In tree-based models, the dataset was iteratively partitioned at each node based on optimized splitting criteria. Various combinations of threshold values were tested to improve performance. For the KNN algorithm, classification was based on the majority group among the K nearest neighbors identified by distance metrics. This non-parametric approach, widely used in supervised learning tasks, relies on instance similarity.

Hyperparameter optimization was performed using grid search cross-validation within the training set to determine the optimal parameters for each algorithm.

Model performance was evaluated using a repeated stratified validation strategy to mitigate the variability associated with small hold-out samples. The dataset was initially partitioned into a fixed independent test set comprising 30% of the data, while the remaining samples were used for model training and hyperparameter optimization. Within the training data, stratified 5-fold cross-validation was applied. This entire procedure was repeated 30 times. Performance metrics, including accuracy, precision, recall, and F1-score, were calculated on the independent test set at each repetition and subsequently summarized as mean ± standard deviation.

## Results

### Sex and age range

In this study, a total of 880 images corresponding to the four PNS were analyzed, with a sex-balanced sample composed of 47.3% males and 52.7% females. Participants ranged in age from 18 to 49 years, with a predominance of young adults, reflected in a mean age of approximately 30 years (Table 1).

**Table 1:** Descriptive statistics, percentage of paranasal sinuses according to sex and age

Age (Years)	Females (*n* = 113)				Males (*n* = 107)				Total (*n* = 220)
	*n* (%)	Mean	Median	SD	*n* (%)	Mean	Median	SD	
18–30	46 (40.7%)	24.0	24.0	3.7	67 (62.6%)	23.1	23.0	3.5	113 (51.4%)
31–49	67 (59.3%)	36.4	35.0	5.4	40 (37.4%)	39.8	40.0	4.7	107 (48.6%)
Total Count	113 (51.4%)	29.6	29.0	7.8	107 (48.6%)	32.3	33.0	9.3	220 (100%)

*SD* standard deviation

### Reliability and reproducibility

No statistically significant differences were observed between the initial measurements and those repeated after a 15-day interval, as determined by the paired t-test. These findings indicate that the data remained stable over time.

Validation of both linear and volumetric measurements yielded excellent reproducibility, with ICC values exceeding 0.800. Additionally, Hotelling’s T² test indicated statistically significant correlation (*p* < 0.001). Two independent assessments of the linear measurements were also performed 15 days apart to evaluate intraobserver reproducibility, further reinforcing the methodological rigor of the protocol.

The results of descriptive statistics and the results of sex estimation with sensitivity, specificity, and AUC ratio summarized in the supplementary material under separate titles as Supplementary material 2 and 3.

### Linear measurements with discriminant analysis

Male individuals exhibited significantly higher mean values in measurements related to the PNS. In the FS, the distances between the anterior and posterior boundaries, as well as between the superior and anterior boundaries, including their corresponding three-dimensional measurements, were statistically greater in males compared to females across all evaluated groups (*p* < 0.05). In the MS, men also demonstrated significantly larger measurements in the superior–inferior dimension on both the right side (*p* < 0.001) and the left side (*p* < 0.009), particularly in the groups from the imaging centers located in Pará and Bahia. These differences were consistent in both planar and three-dimensional measurements. Similarly, greater mean values in the superior–inferior linear dimensions of the SS were also observed in male individuals, both in planar analysis (*p* < 0.003) and in three-dimensional measurements (*p* < 0.008).

### Linear and volumetric measurements with machine learning

Four supervised machine learning algorithms (Random Forest (RF), Logistic Regression (LR), Support Vector Machine (SVM), and K-Nearest Neighbors (KNN)) were evaluated for sex estimation based on linear, three-dimensional, and volumetric PNS measurements. Model performance was assessed using a repeated stratified validation strategy, and classification metrics are summarized in Table 2.

**Table 2:** Performance metrics (mean ± SD) from repeated stratified validation of four machine learning algorithms applied to paranasal sinus measurements, based on selected parameters

Algorithm	Linear measurements				Three-dimensional measurements				Volumetric measurements			
	Accuracy	Precision	Recall	F1-score	Accuracy	Precision	Recall	F1-score	Accuracy	Precision	Recall	F1-score
RF	0.797 ± 0.037	0.800 ± 0.037	0.794 ± 0.037	0.793 ± 0.037	0.698 ± 0.037	0.678 ± 0.037	0.678 ± 0.037	0.695 ± 0.037	0.634 ± 0.037	0.632 ± 0.037	0.632 ± 0.037	0.623 ± 0.037
LR	0.744 ± 0.118	0.748 ± 0.118	0.744 ± 0.118	0.743 ± 0.118	0.837 ± 0.118	0.843 ± 0.118	0.837 ± 0.118	0.836 ± 0.118	0.735 ± 0.118	0.736 ± 0.118	0.735 ± 0.118	0.758 ± 0.118
SVM	0.766 ± 0.105	0.769 ± 0.105	0.766 ± 0.105	0.765 ± 0.105	0.841 ± 0.105	0.838 ± 0.105	0.835 ± 0.105	0.839 ± 0.105	0.742 ± 0.105	0.735 ± 0.105	0.736 ± 0.105	0.738 ± 0.105
KNN	0.742 ± 0.092	0.745 ± 0.092	0.743 ± 0.092	0.741 ± 0.092	0.743 ± 0.092	0.746 ± 0.092	0.742 ± 0.092	0.744 ± 0.092	0.812 ± 0.092	0.795 ± 0.092	0.797 ± 0.092	0.797 ± 0.092

*RF* Random Forest tree, *RL* Logistic regression, *SVM* Support vector machine, *KNN* K-Nearest Neighbors

For linear measurements, RF achieved the highest average accuracy (approximately 0.80), whereas for three-dimensional measurements, both LR and SVM demonstrated superior performance, each reaching mean accuracies of approximately 0.84. These models consistently outperformed the remaining algorithms across repeated evaluations. The final selected hyperparameter configurations for the best-performing models are reported in the Supplementary Material and in Table 3.

**Table 3:** Hyperparameter tuning ranges for each machine learning model

Method	Hyperparameter	Tuning range
Random Forest	n_estimators_range	50,100,150,200,250,300
	criterions	‘gini’, ‘entropy’, ‘log_loss’
	max_depth_range	5,10,15,20
Logistic Regression	C	0.01, 0.1, 1, 10, 100
	Solver	‘liblinear’, ‘saga’
	max_iter	200, 350, 500
	penalty	500, 1000, 2000
Linear SVM	C	0.01, 0.1, 1, 10, 100
	penalty	‘l2’
	loss	‘squared_hinge’
	dual	True, False
	max_iter	500, 1000, 2000
K-Nearest Neighbors	n_neighbors_range	1-130
	p_range	1–6
	metrics_list	‘minkowski’, ‘euclidean’, ‘manhattan’

*RF* Random Forest tree, *LR* Logistic regression, *SVM* Support vector machine, *KNN* K-Nearest Neighbors

Across all measurement types, features related to the frontal sinus (FS) consistently exhibited relevant coefficients and contributions across different algorithms, highlighting their discriminative potential for sex estimation. Among the evaluated classifiers, linear SVM and LR achieved the most robust overall performance, with mean F1-scores exceeding 0.80, indicating a balanced trade-off between precision and recall.

**Fig. 3:**
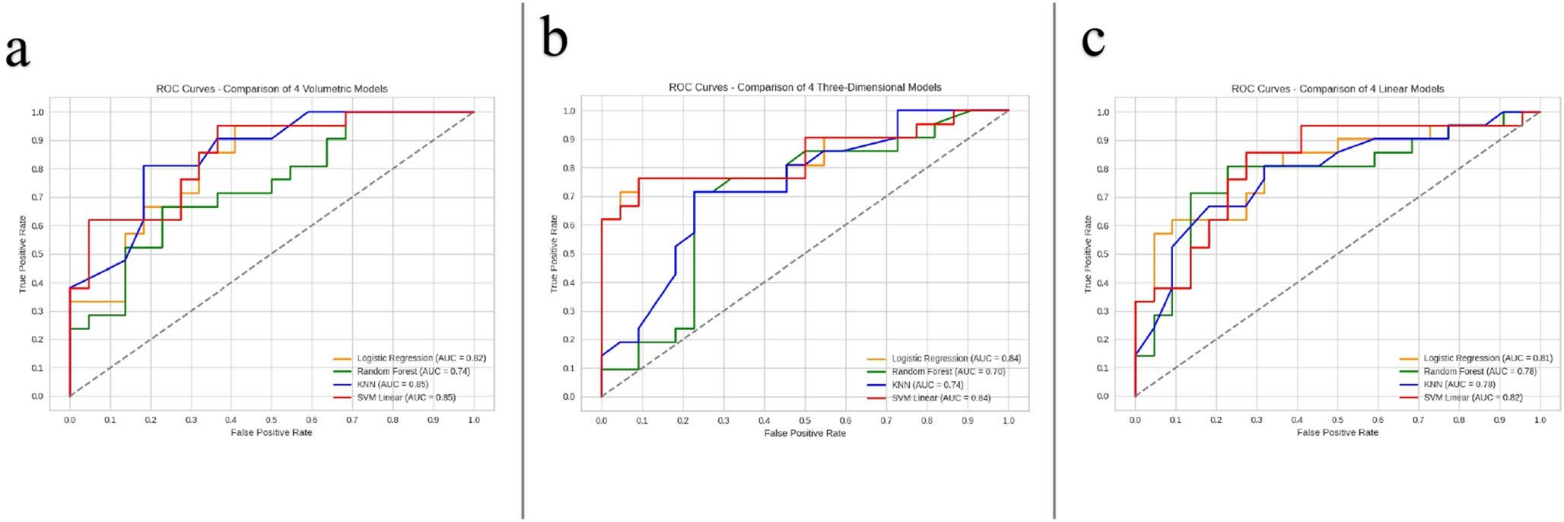
Note. This data is mandatory. Please provide

Overall, weighted average F1-scores ranged from approximately 0.79 to 0.84 across models and measurement strategies (Table 2), supporting the reliability and stability of the proposed machine learning approach for forensic sex estimation. Analyses involving KNN classifiers demonstrated competitive performance in specific scenarios, although with greater variability when compared to LR and SVM models, reinforcing the potential of supervised learning methods for sex estimation based on anatomical structures (Fig. 3).

## Discussion

This study evaluated CT scans of the MS, FS, and SS to investigate the presence of sexual dimorphism. Earlier research has explored this topic using both conventional craniofacial radiography [4, 38, 39] and cone-beam computed tomography (CBCT) [40]. However, studies using CT to develop and validate formulas for sex estimation based on the combined use of linear measurements from the MS and FS remain scarce [20, 23]. Additionally, although variables such as size, area, and total volume have been explored in various investigations, few have examined the direct association between volumetric data and linear measurements [45–48]. To the best of our knowledge, no Brazilian studies have been published using a methodological approach comparable to that adopted in the present work. In this context, we propose an innovative method for assessing the PNS cavities for the purpose of sex estimation, integrating semiautomatic segmentation with specific linear measurements.

An increasing number of studies have explored the identification potential of three-dimensional anatomical structures using quantitative approaches based on point-to-point distance measurements, as observed in the work by De Mendonça et al. [20]. Paranasal sinuses have been shown to exhibit sexual dimorphism in both their linear dimensions and volumetric features. When combined with the widespread use of advanced medical imaging modalities such as CT and CBCT, which enable accurate, non-invasive three-dimensional reconstruction, these anatomical structures emerge as promising candidates for forensic sex estimation [24]. Various methods have been applied in forensic investigations to predict sex based on CT scans, as demonstrated by De Mendonça et al. [25]. Since human bones are inherently three-dimensional structures, analyzing anatomical data in 3D offers increased precision compared to traditional two-dimensional approaches [26, 27].

Historically, the morphology and size of the PNS were assessed by injecting radiopaque materials into cadavers, followed by conventional radiographic imaging [28]. Studies have shown that the PNS often remain preserved even in incinerated remains [29, 30]. Among the sinuses most frequently evaluated are the FS [3, 31–33], SS [34, 35], and MS [2, 7, 36, 37], although the majority of studies have focused on only one type of paranasal sinus.

In contrast, the present study analyzed CT images of the MS, FS, and SS to investigate sexual dimorphism. Previous research employed conventional radiographs of the craniofacial complex [4, 38, 39] and CBCT [40]; however, few studies have used CT to develop and validate sex estimation formulas based on the combination of morphometric (linear distance) measurements from the MS and FS [20, 23].

Volumetric analysis of the FS aimed at individual identification or investigation of sexual dimorphism using CT remains limited [41–44]. This study proposed a method combining semiautomatic segmentation of the FS cavity with specific linear measurements for sex estimation. Although variables such as size, area, and total volume have been independently evaluated in previous research, none have explored the direct association between volume and linear measurements [45–48].

Previous studies have also confirmed sex-related differences in the dimensions of the MS [2, 50, 51], with a consistent tendency for larger values in males, particularly in anteroposterior measurements and volumetric assessments. In females, a discriminant factor of 68.9% was observed based on MS volume. These findings reinforce the utility of CT-derived measurements—especially MS volume—as an auxiliary tool for sex determination in forensic anthropological contexts. In addition, Pearson correlation analysis revealed a weak correlation between age and MS volume, corroborating the findings reported by Wu et al. [49].

Beyond its role in preoperative evaluation, the SS has been extensively studied worldwide due to its applicability in sex estimation of unidentified individuals. The SS is known for its high degree of morphological variability [52, 53], including anatomically relevant variations owing to its proximity to critical neurovascular structures, particularly in the context of transsphenoidal pituitary surgery [54].

This study evaluated linear and volumetric measurements of the PNS in samples from the North and Northeast regions of Brazil. Although the sample was limited to individuals from two geographic regions, this demographic scope holds considerable relevance, as it includes populations that are often underrepresented in forensic studies. Variations in AUC values, sensitivity, and specificity were observed across imaging centers, suggesting that regional and population-specific factors may influence the anatomical characteristics of the PNS. As noted by Vodanović et al. [10], region-specific investigations are critical for developing personalized and more accurate models in both clinical and legal settings. This heterogeneity reinforces the need for regional approaches and multicenter validation, as emphasized by Zolotenkova et al. [55] and Hekimoglu et al. [56], who reported the influence of genetic, ethnic, and environmental factors on craniofacial morphometry. Moreover, Zolotenkova et al. [55] highlighted that the morphological diversity across populations justifies local algorithm validation as a necessary step prior to broader generalization at national or international levels.

In the context of forensic anthropology, the reliability and reproducibility of measurements are essential to ensure that experts can understand and replicate the findings. To achieve this, such measurements must be clearly defined [57]. In the present study, the intraclass correlation coefficient (ICC) exceeded 0.82, demonstrating both high reproducibility and methodological stability, an aspect rarely reported with such a level of detail in AI-based prediction studies. As noted by Niño-Sandoval et al. [15], the standardization of measurement protocols is one of the key factors influencing the reliability of predictive models in forensic anthropology.

Previous studies using MSCT and CBCT, such as those conducted by Oliveira et al. [58] and Ramos et al. [57], also employed ITK-SNAP^®^ 3.0 software to measure SS volume. Ramos et al. [57], in their analysis of 145 females and 123 males aged 18 to 70 years, identified significantly larger volumes in males. In contrast, Oliveira et al. [58], who evaluated 47 scans (27 females and 20 males), found no significant correlations between sex and SS volume. Similarly, in the present study, based on 220 CT scans, no significant association was observed between these variables.

In the present study, machine learning models were used to process large volumes of data derived from tomographic images, allowing for faster and more precise sex estimation, consistent with previous studies applying deep learning to craniofacial imaging [59]. Although independent data partitions were employed for training and testing, previous studies, such as those by Demir et al. [60] and Patil et al. [17], have demonstrated that stratified cross-validation, particularly using the k-fold method, enhances the robustness of predictive outcomes, mitigates overfitting, and optimizes sample utilization. Future research could benefit from the implementation of such techniques, which would also allow the extraction of confidence intervals for performance metrics.

The use of semiautomated tools such as ITK-SNAP and 3D Slicer was chosen due to their wide acceptance in the literature and their ability to provide detailed control over the anatomical region of interest. This approach was preferred over fully automated segmentation to ensure precision during dataset construction, which is a critical step in AI applications within healthcare. As noted by Niño-Sandoval et al. [15], this manual curation contributes to the integrity of the data used in machine learning models. Accordingly, the adoption of machine learning was a deliberate choice aimed at enhancing interpretability and control over input parameters, which is particularly important in forensic contexts. As demonstrated by Patil et al. [17], the use of machine learning models based on derived morphometric variables enables effective and interpretable modeling, especially useful in legal settings where model explainability is crucial.

The decision to use derived measurements (volumes and distances) as input for AI models allowed for greater control over the variables of interest and improved interpretability of the results. This approach is recommended in forensic scenarios, where transparency and explainability are essential for decision-making. However, as shown by Vila-Blanco et al. [18] and Kim et al. [61], end-to-end models that process raw CT images can capture more subtle morphological patterns and improve predictive performance. The integration of derived variables with features extracted directly by convolutional neural networks may represent a promising advancement.

Among the three sets of variables analyzed, the three-dimensional dataset resulted in the best overall performance across the algorithms. This was consistently observed in both F1-score and accuracy metrics, particularly for classifiers based on support vector machines (SVM) and logistic regression (LR). These findings corroborate previous studies identifying three-dimensional variables as highly informative for sex discrimination based on craniofacial structures [15, 60, 62]. In general, the models demonstrated higher recall for male individuals and greater precision for female individuals (recall of 0.91 for males and a precision of 0.89 for females). This pattern suggests that the models are more sensitive in identifying male individuals, while tending to be more conservative in classifying females—an important consideration for forensic applications.

The evaluation of multiple machine learning algorithms, including SVM and logistic regression (LR), enabled a comprehensive comparative analysis of predictive methods. Our findings demonstrated variability in predictive performance across algorithms, with LR and linear SVM emerging as the effective across all three variable sets (linear, three-dimensional, and volumetric). These results are consistent with those reported by Demir et al. [60], who highlighted the superior performance of neural networks and SVMs in sex estimation using digital cranial images. The assessment of different model architectures aligns with trends observed in recent forensic studies, such as those reported by Hekimoglu et al. [56], which emphasize the importance of testing diverse models to maximize diagnostic accuracy. Moreover, the combination of different variable sets (i.e., hybrid models) improved F1-scores, indicating a balanced trade-off between precision and recall, which is an especially critical metric in forensic applications where both false positives and false negatives carry substantial implications. Future studies are encouraged to perform more refined hyperparameter optimization using strategies such as GridSearch or Bayesian Optimization, along with comprehensive reporting of performance metrics, including F1-score, precision, and ROC curve analysis.

The results also showed that three-dimensional measurements of the FS and MS achieved robust AUC values (greater than 0.70 in various combinations), indicating strong discriminatory potential. According to Zolotenkova et al. [55], AUC values between 0.70 and 0.80 are considered acceptable in forensic applications, provided that measurement protocols are highly standardized. These findings thus support the feasibility of using such variables for sex estimation with scientific reliability and forensic applicability. The 84% accuracy achieved by the machine learning models in this study exceeds the average values reported by Patil et al. [17], who used mandibular morphometric variables and obtained accuracies ranging from 72% to 81%. Similarly, Vodanović et al. [10] reported an 81% accuracy in sex prediction based on dental CT scans of Croatian adults, suggesting that the models applied in this study are in line with the expected performance of supervised algorithms in medical imaging.

Some limitations of this study should be acknowledged. All measurements were performed by a single observer; although prior calibration and intraobserver agreement assessment indicated temporal stability, the inclusion of an independent second observer would further strengthen measurement robustness. In addition, the restricted age range of the sample (18–49 years), adopted to minimize the influence of age-related skeletal changes, limits the applicability of the proposed models to older adults, who frequently represent an important demographic in forensic identification contexts.

Although ITK-SNAP and 3D Slicer are validated tools for segmentation, the measurement process is complex, and semiautomated methods may be affected by under- or over-segmentation errors, which can reduce operational efficiency. Future investigations employing fully automated, AI-assisted segmentation methods should be considered to improve reproducibility and measurement accuracy. Furthermore, it must be recognized that artificial intelligence does not replace human expertise, but rather serves as a complementary tool. Clinical judgment and professional experience remain irreplaceable, especially in complex cases that require critical interpretation. It is also worth noting that AI is an evolving technology, whose accuracy depends directly on the quality of the training data and the specificity of the application context.

Recent advances in machine learning and computer vision have substantially expanded the scope of forensic anthropology. Paranasal sinuses have emerged as particularly relevant anatomical structures due to their complex and individualized morphology, which supports applications in sex estimation, age prediction, and personal identification. A recent comprehensive survey by Alsalama et al. [63] systematically reviewed machine learning and deep learning approaches applied to paranasal sinus analysis, reporting consistently high performance across different forensic tasks. Within this evolving methodological landscape, the present study contributes by evaluating morphometric features derived from three-dimensional imaging in a multicenter context, providing additional evidence on the applicability and generalizability of machine learning–based approaches.

The findings of the present study revealed anatomical variability of the MS between sexes. Statistically significant differences were observed in MS volumes between males and females on both sides, indicating sexual dimorphism, particularly in the FS and MS. Although machine learning models, especially linear SVM and logistic regression, which achieved accuracies of up to 84% in sex estimation. The use of supervised learning algorithms proved effective when applied to tomographic imaging data, reinforcing the potential of artificial intelligence as a valuable complementary tool in forensic identification practices.

## Supplementary Information

Below is the link to the electronic supplementary material.

Supplementary Material 1 (DOCX 63.5 KB)

## Declarations

### Generative AI in scientific writing

During the preparation of this work, we did not use of generative AI and AI-assisted technologies in the writing process.
