# Supplementary material for: Development of an AI-based model for sex estimation using CT-derived metrics from paranasal sinuses
Source: Int J Legal Med. 2026 Apr 6;140(4):2383–94. doi: 10.1007/s00414-026-03789-y (PMC13275773; doi:10.1007/s00414-026-03789-y)
Supplement: Supplementary file 1 — Supplementary Material 1 (DOCX 63.5 KB) [file 414_2026_3789_MOESM1_ESM.docx]

**Supplementary material 1**

Table. Definition of the linear parameters used for the assessment of the paranasal sinuses.

| **Paranasal sinus** | **Measurement (mm)** | **Reference points** | **Figure 2** |
| --- | --- | --- | --- |
| Frontal | Supero-inferior, anteroposterior, latero-medial, supero-left, supero-right, antero-right, antero-left, supero-anterior | SFS, IFS, AFS, PFS, RFS, LFS | a, b, c |
| Maxillary | Supero-inferior, anteroposterior, latero-medial | SRMS, IRMS, ARMS, PRMS, RLRMS, LLRMS, SLMS, ILMS, ALMS, PLMS, RLLMS, LLLMS, | d, e, f |
| Sphenoidal | Supero-inferior, anteroposterior, latero-medial | SSS, ISS, ASS, PSS, LSS, RSS | g, h, i |

SFS: superior frontal sinus point; IFS, inferior frontal sinus point; AFS, Anterior frontal sinus; PFS, Posterior frontal sinus; LFS, left frontal sinus; RFS, right frontal sinus; SRMS, superior right maxillary sinus; IRMS, inferior right maxillary sinus; ARMS, anterior right maxillary sinus; PRMS, posterior right maxillary sinus; RLRMS, right lateral point of the right maxillary sinus; LLRMS, left lateral point of the right maxillary sinus; SLMS, Superior left maxillary sinus; ILMS, Inferior left maxillary sinus; ALMS, Anterior left maxillary sinus; PLMS, Posterior left maxillary sinus; RLLMS, right lateral point of the lef maxillary sinus; LLLMS, left lateral point of the lef maxillary sinus; SSS, superior sphenoidal sinus; ISS, inferior sphenoidal sinus; RSS, right sphenoidal sinus; LSS, left sphenoidal sinus; PSS, posterior sphenoidal sinus, and ASS, anterior sphenoidal sinus.

**Supplementary material 2**

Summary of sensitivity, specificity, and AUC ratio for paranasal sinuses variables used in sex estimation.

Table. Summary of sensitivity, specificity, and AUC ratio for frontal sinus (F) variables used in sex estimation.

|  | **Imaging Center 1 (Pará)** | | | | | |  | **Imaging Center 2 (Bahia)** | | | | | |  | **Imaging Center 3 (Ceará)** | | | | | | |
| --- | --- | --- | --- | --- | --- | --- | --- | --- | --- | --- | --- | --- | --- | --- | --- | --- | --- | --- | --- | --- | --- |
| **Variables (Measurement direction)** | **p-value** | **AUC±SEM (CI95%)** |  | **Cutoff** | **Sen** | **Spec** |  | **p-value** | **AUC±SEM (CI95%)** |  | **Cutoff** | **Sen** | **Spec** |  | **p-value** | **AUC±SEM (CI95%)** |  | **Cutoff** | **Sen** | **Spec** |  |
| F - S-I (a) | **0.003** | 0.666±0.053(0.562-0.770) | > | -33.37 | 64.8% | 64.8% |  | 0.333 | 0.583±0.091(0.406-0.761) | > | -33.03 | 60.4% | 60.4% |  | 0.178 | 0.629±0.086(0.460-0.798) | > | -31.71 | 66.7% | 66.7% |  |
| F - S-I (b) | **0.008** | 0.651±0.054(0.546-0.757) | < | 36.13 | 63.0% | 37.0% |  | 0.055 | 0.665±0.076(0.516-0.814) | < | 34.44 | 58.3% | 41.7% |  | 0.118 | 0.650±0.088(0.478-0.822) | < | 32.95 | 66.7% | 33.3% |  |
| F - A-P (c) | **0.000** | 0.784±0.045(0.696-0.872) | > | -20.40 | 70.4% | 70.4% |  | **0.011** | 0.719±0.074(0.574-0.864) | > | -19.99 | 60.4% | 60.4% |  | 0.193 | 0.625±0.085(0.458-0.792) | > | -18.12 | 58.3% | 58.3% |  |
| F - A-P (b) | **0.002** | 0.676±0.053(0.573-0.779) | < | 27.19 | 57.4% | 42.6% |  | **0.003** | 0.751±0.068(0.618-0.884) | < | 29.47 | 70.8% | 29.2% |  | **0.048** | 0.690±0.082(0.529-0.850) | < | 24.69 | 66.7% | 33.3% |  |
| F - L-R (d) | **0.010** | 0.645±0.054(0.540-0.751) | < | 55.37 | 61.1% | 38.9% |  | 0.175 | 0.617±0.093(0.434-0.799) | < | 53.10 | 60.4% | 39.6% |  | 0.224 | 0.617±0.089(0.442-0.791) | < | 54.72 | 58.3% | 41.7% |  |
| F - L-R (b) | **0.009** | 0.647±0.054(0.542-0.753) | < | 55.43 | 61.1% | 38.9% |  | 0.053 | 0.667±0.084(0.502-0.832) | < | 55.19 | 66.7% | 33.3% |  | 0.224 | 0.617±0.089(0.442-0.791) | < | 54.90 | 66.7% | 33.3% |  |
| F - S-L (d) | 0.151 | 0.581±0.056(0.472-0.691) | > | -26.71 | 53.7% | 53.7% |  | 0.057 | 0.664±0.077(0.512-0.816) | > | -27.90 | 60.4% | 60.4% |  | 0.409 | 0.579±0.094(0.395-0.763) | < | 54.77 | 58.3% | 41,7% |  |
| F - S-L (b) | 0.058 | 0.607±0.055(0.499-0.716) | < | 30.92 | 57.4% | 42.6% |  | 0.055 | 0.665±0.079(0.511-0.819) | < | 31.99 | 60.4% | 39.6% |  | 0.268 | 0.606±0.090(0.429-0.783) | < | 54.94 | 66.7% | 33.3% |  |
| F - S-R (d) | **0.024** | 0.627±0.055(0.519-0.735) | < | 28.38 | 57.4% | 42.6% |  | 0.160 | 0.621±0.096(0.432-0.809) | < | 25.84 | 56.3% | 43.8% |  | 0.385 | 0.583±0.087(0.413-0.753) | > | -28.67 | 58.3% | 58.3% |  |
| F - S-R (b) | **0.005** | 0.659±0.054(0.553-0.765) | < | 33.95 | 64.8% | 35.2% |  | 0.116 | 0.635±0.088(0.462-0.808) | < | 30.74 | 60.4% | 39.6% |  | 0.328 | 0.594±0.087(0.423-0.764) | < | 33.82 | 66.7% | 33.3% |  |
| F - A-L (d) | **0.015** | 0.638±0.055(0.531-0.745) | > | -26.72 | 59.3% | 59.3% |  | **0.010** | 0.721±0.079(0.566-0.876) | > | -27.87 | 66.7% | 66.7% |  | 0.200 | 0.623±0.089(0.449-0.797) | < | 25.75 | 58.3% | 41.7% |  |
| F - A-L (b) | **0.008** | 0.650±0.054(0.544-0.756) | < | 29.89 | 61.1% | 38.9% |  | **0.008** | 0.728±0.075(0.581-0.874) | < | 30.57 | 66.7% | 33.3% |  | 0.140 | 0.642±0.088(0.469-0.814) | < | 33.30 | 50.0% | 50.0% |  |
| F - A-R (d) | 0.207 | 0.572±0.056(0.461-0.682) | < | 27.03 | 55.6% | 44.4% |  | 0.498 | 0.558±0.096(0.371-0.746) | < | 26,88 | 54.2% | 45.8% |  | 0.617 | 0.548±0.085(0.382-0.714) | > | -27.99 | 58.3% | 58.3% |  |
| F - A-R (b) | 0.087 | 0.597±0.056(0.487-0.706) | < | 30.14 | 57.4% | 42.6% |  | 0.317 | 0.586±0.091(0.407-0.765) | < | 28.57 | 54.2% | 45.8% |  | 0.487 | 0.567±0.084(0.401-0.732) | < | 30.60 | 58.3% | 41.7% |  |
| F - S-A (a) | **0.000** | 0.751±0.047(0.658-0.844) | > | -16.64 | 66.7% | 66.7% |  | **0.012** | 0.715±0.078(0.562-0.869) | > | -16.23 | 66.7% | 66.7% |  | 0.108 | 0.654±0.086(0.485-0.823) | > | -17.31 | 66.7% | 66.7% |  |
| F - S-A (b) | **0.000** | 0.765±0.047(0.673-0.857) | < | 20.49 | 70.4% | 29.6% |  | **0.001** | 0.776±0.063(0.652-0.901) | < | 18.49 | 72.9% | 27.1% |  | **0.016** | 0.731±0.086(0.563-0.900) | < | 18.40 | 66.7% | 33.3% |  |
| F - Volume | **0.000** | 0.755±0.047(0.663-0.846) | < | 7202.61 | 68.5% | 31.5% |  | **0.007** | 0.731±0.078(0.579-0.882) | < | 6064.16 | 66.7% | 33.3% |  | 0.094 | 0.660±0.082(0.499-0.822) | < | 6068.68 | 66.7% | 33.3% |  |

A: Anterior; AUC: Area under the curve; CI: Confidence interval; S: superior; I: Inferior; L: Left; R: right; P: posterior; R: Right; Sens: Sensitivity; S: Superior; Spec: Specificity.

Measurement directions: a (linear measurement from the superior to the inferior aspect); b (tridimensional measurement), c (linear measurement from the anterior to the posterior aspect), and d (linear measurement from the right to the left aspect).

Table. Summary of sensitivity, specificity, and AUC ratio for right (RM) and left (LM) maxillary sinus variables used in sex estimation.

|  | **Imaging Center 1 (Pará)** | | | | | |  | **Imaging Center 2 (Bahia)** | | | | | |  | | **Imaging Center 3 (Ceará)** | | | | | | |
| --- | --- | --- | --- | --- | --- | --- | --- | --- | --- | --- | --- | --- | --- | --- | --- | --- | --- | --- | --- | --- | --- | --- |
| **Variables (Measurement direction)** | **p-value** | **AUC±SEM (CI95%)** |  | **Cutoff** | **Sen** | **Spec** |  | **p-value** | **AUC±SEM (CI95%)** |  | **Cutoff** | **Sen** | **Spe** |  | **p-value** | | **AUC±SEM (CI95%)** |  | **Cutoff** | **Sen** | **Spec** |  |
| RM - S-I (a) | **0.000** | 0.750±0.049(0.655-0.845) | > | -40.10 | 74.1% | 74.1% |  | **0.001** | 0.776±0.059(0.660-0.893) | > | -40.70 | 64.6% | 64.6% |  | 0.193 | | 0.625±0.103(0.423-0.827) | > | -37.17 | 58.3% | 58.3% |  |
| RM - S-I (b) | **0.000** | 0.730±0.050(0.631-0.828) | < | 41.46 | 72.2% | 27.8% |  | **0.008** | 0.729±0.068(0.595-0.863) | < | 41.91 | 60.4% | 39.6% |  | 0.103 | | 0.656±0.104(0.452-0.860) | < | 39.81 | 58.3% | 41.7% |  |
| RM - A-P (c) | **0.006** | 0.657±0.054(0.552-0.762) | > | -40.17 | 61.1% | 61.1% |  | 0.411 | 0.571±0.081(0.411-0.731) | > | -40.16 | 58.3% | 58.3% |  | **0.023** | | 0.719±0.095(0.532-0.905) | > | -38.03 | 66.7% | 66.7% |  |
| RM - A-P (b) | **0.001** | 0.694±0.052(0.592-0.796) | < | 41.19 | 63.0% | 37.0% |  | 0.458 | 0.564±0.081(0.405-0.723) | < | 40.96 | 52.1% | 47.9% |  | **0.012** | | 0.742±0.092(0.561-0.922) | < | 38.74 | 75.0% | 25.0% |  |
| RM - L-R (d) | 0.489 | 0.539±0.057(0.427-0.651) | > | 32.85 | 51.9% | 51.9% |  | 0.208 | 0.608±0.077(0.458-0.758) | < | 31.95 | 62.5% | 37.5% |  | 0.193 | | 0.625±0.095(0.440-0.810) | < | 31.33 | 58.3% | 41.7% |  |
| RM - R-L (b) | 0.281 | 0.561±0.058(0.448-0.674) | < | 35.91 | 53.7% | 46.3% |  | 0.073 | 0.654±0.074(0.510-0.798) | < | 35.89 | 64.6% | 35.4% |  | 0.103 | | 0.656±0.090(0.479-0.833) | < | 33.85 | 66.7% | 33.3% |  |
| RM - Volume | **0.003** | 0.671±0.053(0.568-0.774) | < | 17441.12 | 63.0% | 37.0% |  | 0.063 | 0.660±0.081(0.500-0.819) | < | 17106.74 | 60.4% | 39.6% |  | 0.090 | | 0.663±0.086(0.493-0.832) | < | 15021.55 | 66.7% | 33.3% |  |
| LM - S-I (a) | **0.000** | 0.722±0.049(0.625-0.819) | > | -39.48 | 66.7% | 66.7% |  | **0.003** | 0.754±0.063(0.631-0.878) | > | -40.59 | 66.7% | 66.7% |  | 0.318 | | 0.596±0.094(0.412-0.779) | < | -38.83 | 50.0% | 50.0% |  |
| LM - S-I (b) | **0.001** | 0.686±0.051(0.585-0.786) | < | 41.80 | 59.3% | 40.7% |  | **0.009** | 0.726±0.070(0.590-0.863) | < | 41.90 | 66.7% | 33.3% |  | 0.152 | | 0.638±0.094(0.453-0.822) | < | 39.86 | 58.3% | 41.7% |  |
| LM - A-P (c) | **0.001** | 0.685±0.052(0.584-0.786) | > | -40.01 | 59.3% | 59.3% |  | 0.098 | 0.642±0.073(0.499-0.786) | > | -40.42 | 62.5% | 62.5% |  | **0.019** | | 0.725±0.088(0.553-0.897) | > | -37.71 | 75.0% | 75.0% |  |
| LM - A-P (b) | **0.002** | 0.680±0.052(0.578-0.781) | < | 40.97 | 59.3% | 40.7% |  | 0.266 | 0.596±0.076(0.447-0.745) | < | 40.98 | 54.2% | 45.8% |  | **0.016** | | 0.731±0.091(0.553-0.910) | < | 38.89 | 75.0% | 25.0% |  |
| LM - L-R (d) | 0.306 | 0.558±0.057(0.447-0.669) | < | 32.63 | 53.7% | 46.3% |  | 0.197 | 0.611±0.078(0.457-0.765) | < | 31.59 | 54.2% | 45.8% |  | 0.103 | | 0.656±0.093(0.474-0.839) | < | 29.51 | 66.7% | 33.3% |  |
| LM - L-R (b) | 0.063 | 0.605±0.056(0.494-0.716) | < | 36.06 | 55.6% | 44.4% |  | 0.197 | 0.611±0.078(0.459-0.764) | < | 35.25 | 58.3% | 41.7% |  | 0.077 | | 0.670±0.087(0.500-0.839) | < | 31.92 | 58.3% | 41.7% |  |
| LM - Volume | **0.001** | 0.682±0.053(0.579-0.785) | < | 17670.88 | 63.0% | 37.0% |  | **0.025** | 0.693±0.075(0.547-0.839) | < | 18202.33 | 64.6% | 35.4% |  | 0.193 | | 0.625±0.093(0.443-0.807) | < | 15145.42 | 58.3% | 41.7% |  |

A: Anterior; AUC: Area under the curve; CI: Confidence interval; S: superior; I: Inferior; L: Left; R: right; P: posterior; R: Right; Sens: Sensitivity; S: Superior; Spec: Specificity.

Measurement directions: a (linear measurement from the superior to the inferior aspect); b (tridimensional measurement), c (linear measurement from the anterior to the posterior aspect), and d (linear measurement from the right to the left aspect).

Table. Summary of sensitivity, specificity, and AUC ratio for sphenoidal (Sph) sinus variables used in sex estimation.

|  | **Imaging Center 1 (Pará)** | | | | | |  | **Imaging Center 2 (Bahia)** | | | | | |  | **Imaging Center 3 (Ceará)** | | | | | |
| --- | --- | --- | --- | --- | --- | --- | --- | --- | --- | --- | --- | --- | --- | --- | --- | --- | --- | --- | --- | --- |
| **Variables (Measurement direction)** | **p-value** | **AUC±SEM (CI95%)** |  | **Cutoff** | **Sen** | **Spec** |  | **p-value** | **AUC±SEM (CI95%)** |  | **Cutoff** | **Sen** | **Spe** |  | **p-value** | **AUC±SEM (CI95%)** |  | **Cutoff** | **Sen** | **Spec** |
| Sph - S-I (a) | **0.005** | 0.660±0.054(0.555-0.766) | > | -26.57 | 64.8% | 64.8% |  | 0.175 | 0.617±0.081(0.458-0.775) | > | -27.21 | 60.4% | 60.4% |  | 0.501 | 0.565±0.099(0.370-0.759) | > | -24.21 | 58.3% | 58.3% |
| Sph - S-I (b) | **0.001** | 0.683±0.053(0.578-0.787) | < | 30.85 | 66.7% | 33.3% |  | 0.180 | 0.615±0.081(0.457-0.774) | < | 32.03 | 62.5% | 37.5% |  | 0.339 | 0.592±0.101(0.394-0.790) | < | 26.93 | 58.3% | 41.7% |
| Sph - A-P (c) | 0.092 | 0.595±0.056(0.487-0.704) | > | -32.85 | 59.3% | 59.3% |  | 0.448 | 0.565±0.084(0.402-0.729) | > | -32.07 | 54.2% | 54.2% |  | 0.099 | 0.658±0.091(0.479-0.837) | > | -30.56 | 66.7% | 66.7% |
| Sph - A-P (b) | **0.019** | 0.633±0.054(0.527-0.740) | < | 35.09 | 59.3% | 40.7% |  | 0.529 | 0.554±0.080(0.397-0.712) | < | 33.38 | 54.2% | 45.8% |  | 0.051 | 0.688±0.090(0.512-0.863) | < | 31.90 | 66.7% | 33.3% |
| Sph - L-R (d) | 0.308 | 0.558±0.057(0.447-0.669) | < | 40.84 | 55.6% | 44.4% |  | 0.084 | 0.649±0.077(0.499-0.799) | < | 40.20 | 60.4% | 39.6% |  | 0.200 | 0.623±0.103(0.420-0.825) | < | 38.17 | 58.3% | 41.7% |
| Sph - L-R (b) | 0.153 | 0.581±0.057(0.470-0.692) | < | 42.28 | 55.6% | 44.4% |  | **0.045** | 0.672±0.072(0.530-0.814) | < | 40.79 | 64.6% | 35.4% |  | 0.216 | 0.619±0.102(0.418-0.819) | < | 38.80 | 58.3% | 41.7% |
| Sph - Volume | **0.037** | 0.618±0.055(0.510-0.726) | < | 10578.41 | 61.1% | 38.9% |  | 0.156 | 0.622±0.082(0.462-0.782) | < | 11397.73 | 60.4% | 39.6% |  | 0.250 | 0.610±0.088(0.438-0.783) | < | 8153.23 | 58.3% | 41.7% |

A: Anterior; AUC: Area under the curve; CI: Confidence interval; S: superior; I: Inferior; L: Left; R: right; P: posterior; R: Right; Sens: Sensitivity; S: Superior; Spec: Specificity.

Measurement directions: a (linear measurement from the superior to the inferior aspect); b (tridimensional measurement), c (linear measurement from the anterior to the posterior aspect), and d (linear measurement from the right to the left aspect).

**Supplementary material 3**

Comparison of the paranasal sinuses’ measurements by side.

| **Paranasal Sinus** | **Measurement** | **Mean** | **Median** | **SD** | **Variance** | **Minimum** | **Maximum** | **Shapiro–Wilk p** |
| --- | --- | --- | --- | --- | --- | --- | --- | --- |
| **Frontal** | S-I | 33.1715 | 33.032 | 10.2905 | 105.8945 | 11.827 | 63.213 | 0.029671 |
|  | A-P | 19.6865 | 19.074 | 5.7862 | 33.4804 | 0.998 | 41.363 | 0.004336 |
|  | L-R | 54.7531 | 54.636 | 17.0869 | 291.9622 | 1.592 | 90.397 | 0.166148 |
|  | S-L | 27.3809 | 27.171 | 11.0136 | 121.3012 | 3.278 | 56.750 | 0.366874 |
|  | S-R | 27.2239 | 26.818 | 11.1034 | 123.2866 | 2.336 | 59.947 | 0.414545 |
|  | A-L | 27.8947 | 26.952 | 10.6290 | 112.9755 | 3.365 | 58.224 | 0.269057 |
|  | A-R | 27.2817 | 27.013 | 9.8641 | 97.3018 | 3.826 | 54.570 | 0.626985 |
|  | S-A | 17.2667 | 16.492 | 10.1060 | 102.1319 | 1.468 | 47.583 | 5.06E-06 |
|  | S-I (3D) | 35.4958 | 34.692 | 9.7060 | 94.2066 | 13.120 | 64.556 | 0.037687 |
|  | A-P (3D) | 28.4283 | 27.132 | 9.0041 | 81.0747 | 10.418 | 60.576 | 8.71E-08 |
|  | L-R (3D) | 55.2604 | 54.828 | 16.6478 | 277.1498 | 9.638 | 90.744 | 0.157085 |
|  | S-L (3D) | 32.8851 | 31.812 | 12.7150 | 161.6723 | 5.301 | 63.704 | 0.126612 |
|  | S-R (3D) | 33.2388 | 33.174 | 12.5599 | 157.7512 | 3.384 | 68.941 | 0.330152 |
|  | A-L (3D) | 30.7336 | 30.226 | 11.4108 | 130.2068 | 5.702 | 63.262 | 0.262151 |
|  | A-R (3D) | 29.9604 | 29.054 | 10.5853 | 112.0499 | 6.007 | 57.088 | 0.210546 |
|  | S-A (3D) | 20.6165 | 18.809 | 11.0123 | 121.2723 | 2.494 | 54.489 | 0.000104 |
|  | Volume | 8166.334 | 6418.755 | 6337.1560 | 40159549 | 261.680 | 31673.340 | 3.39E-12 |
| **Right maxillary** | S-I | 39.3280 | 39.584 | 5.3022 | 28.1142 | 10.695 | 52.748 | 2.08E-05 |
|  | A-P | 39.5844 | 39.923 | 3.7356 | 13.9551 | 10.492 | 49.716 | 1.55E-12 |
|  | L-R | 31.6269 | 31.940 | 4.9302 | 24.3077 | 9.609 | 48.891 | 1.69E-05 |
|  | S-I (3D) | 41.0839 | 41.371 | 5.4623 | 29.8373 | 12.680 | 55.427 | 2.23E-05 |
|  | A-P (3D) | 40.7033 | 40.626 | 3.8148 | 14.5529 | 23.381 | 57.185 | 0.000165 |
|  | R-L (3D) | 35.0659 | 35.488 | 5.2670 | 27.7423 | 12.052 | 53.872 | 2.06E-05 |
|  | Volume | 98906.24 | 16902.39 | 1214992 | 1.48E+12 | 27.696 | 18038070 | 9.81E-32 |
| **Left maxillary** | S-I | 39.4455 | 39.336 | 4.9166 | 24.1730 | 26.073 | 52.620 | 0.716600 |
|  | A-P | 39.5261 | 39.739 | 3.1832 | 10.1333 | 28.477 | 48.778 | 0.118751 |
|  | L-R | 31.7323 | 31.943 | 4.5292 | 20.5143 | 19.630 | 48.432 | 0.003198 |
|  | S-I (3D) | 41.2512 | 41.011 | 5.0666 | 25.6712 | 27.489 | 55.908 | 0.817242 |
|  | A-P (3D) | 40.6543 | 40.718 | 3.7288 | 13.9039 | 29.850 | 53.018 | 0.026692 |
|  | L-R (3D) | 35.3607 | 35.443 | 4.9146 | 24.1538 | 22.584 | 54.648 | 0.001755 |
|  | Volume | 17308.63 | 17218.73 | 5212.3510 | 27168602 | 6483.600 | 33455.220 | 0.001963 |
| **Sphenoid** | S-I | 26.4224 | 26.290 | 5.0737 | 25.7429 | 13.895 | 44.468 | 0.165516 |
|  | A-P | 31.5066 | 32.054 | 5.7502 | 33.0650 | 13.353 | 43.975 | 0.000700 |
|  | L-R | 40.4774 | 39.866 | 9.5217 | 90.6640 | 17.465 | 70.815 | 0.001849 |
|  | S-I (3D) | 31.3658 | 30.520 | 7.5345 | 56.7695 | 15.193 | 53.687 | 3.91E-05 |
|  | A-P (3D) | 33.9504 | 33.684 | 6.4007 | 40.9692 | 15.548 | 55.070 | 0.667458 |
|  | L-R (3D) | 41.2740 | 40.851 | 9.4856 | 89.9777 | 19.397 | 71.1720 | 0.002115 |
|  | Volume | 10530.05 | 9971.58 | 4952.8390 | 24530617 | 1223.880 | 30396.950 | 6.21E-06 |

S-I: superior and inferior planes distance; A-P anterior and posterior planes distance; L-R-: left and right planes distance; S-L-: superior and left planes distance; S-R-: superior and right planes distance; A-L-: anterior and left planes distance; A-R-: anterior and right planes distance; S-A: superior and anterior planes distance; S-I (3D): superior and inferior points distance; A-P (3D) anterior and posterior points distance; L-R (3D)-: left and right points distance; S-L (3D)-: superior and left points distance; S-R (3D)-: superior and right points distance; A-L (3D)-: anterior and left points distance; A-R (3D)-: anterior and right points distance; S-A (3D): superior and anterior points distance.

Table. Area under the ROC curve (AUC) values stratified by imaging center.

| **Paranasal Sinus** | **Measurement** | **p-** | **Pará** | **p-** | **Bahia** | **p-** | **Ceará** |
| --- | --- | --- | --- | --- | --- | --- | --- |
|  |  | **Value** | **AUC (IC95%)** | **Value** | **AUC (IC95%)** | **Value** | **AUC (IC95%)** |
| Frontal | S-I | **0.003** | **0.666±0.053(0.562-0.770)** | 0.333 | 0.583±0.091(0.406-0.761) | 0.178 | 0.629±0.086(0.460-0.798) |
|  | S-I (3D) | **0.008** | **0.349±0.054(0.243-0.454)** | 0.055 | 0.335±0.076(0.186-0.484) | 0.118 | 0.350±0.088(0.178-0.522) |
|  | A-P | **0.000** | **0.784±0.045(0.696-0.872)** | **0.011** | **0.719±0.074(0.574-0.864)** | 0.193 | 0.625±0.085(0.458-0.792) |
|  | A-P (3D) | **0.002** | **0.324±0.053(0.221-0.427)** | **0.003** | **0.249±0.068(0.116-0.382)** | 0.048 | 0.310±0.082(0.150-0.471) |
|  | L-R | **0.010** | **0.355±0.054(0.249-0.460)** | 0.175 | 0.383±0.093(0.201-0.566) | 0.224 | 0.383±0.089(0.209-0.558) |
|  | L-R (3D) | **0.009** | **0.353±0.054(0.247-0.458)** | 0.053 | 0.333±0.084(0.168-0.498) | 0.224 | 0.383±0.089(0.209-0.558) |
|  | S-L | 0.151 | 0.581±0.056(0.472-0.691) | 0.057 | 0.664±0.077(0.512-0.816) | 0.409 | 0.579±0.094(0.395-0.763) |
|  | S-L (3D) | 0.058 | 0.393±0.055(0.284-0.501) | 0.055 | 0.335±0.079(0.181-0.489) | 0.268 | 0.394±0.090(0.217-0.571) |
|  | S-R | **0.024** | **0.373±0.055(0.265-0.481)** | 0.160 | 0.379±0.096(0.191-0.568) | 0.385 | 0.417±0.087(0.247-0.587) |
|  | S-R (3D) | **0.005** | **0.341±0.054(0.235-0.447)** | 0.116 | 0.365±0.088(0.192-0.538) | 0.328 | 0.406±0.087(0.236-0.577) |
|  | A-L | **0.015** | **0.638±0.055(0.531-0.745)** | **0.010** | **0.721±0.079(0.566-0.876)** | 0.200 | 0.623±0.089(0.449-0.797) |
|  | A-L (3D) | **0.008** | **0.350±0.054(0.244-0.456)** | **0.008** | **0.272±0.075(0.126-0.419)** | 0.140 | 0.358±0.088(0.186-0.531) |
|  | A-R | 0.207 | 0.428±0.056(0.318-0.539) | 0.498 | 0.442±0.096(0.254-0.629) | 0.617 | 0.452±0.085(0.286-0.618) |
|  | A-R (3D) | 0.087 | 0.403±0.056(0.294-0.513) | 0.317 | 0.414±0.091(0.235-0.593) | 0.487 | 0.433±0.084(0.268-0.599) |
|  | S-A | **0.000** | **0.751±0.047(0.658-0.844)** | **0.012** | **0.715±0.078(0.562-0.869)** | 0.108 | 0.654±0.086(0.485-0.823) |
|  | S-A (3D) | **0.000** | **0.235±0.047(0.143-0.327)** | **0.001** | **0.224±0.063(0.099-0.348)** | **0.016** | **0.269±0.086(0.100-0.437)** |
|  | Volume | **0.000** | **0.245±0.047(0.154-0.337)** | **0.007** | **0.269±0.078(0.118-0.421)** | 0.094 | 0.340±0.082(0.178-0.501) |
| Right maxillary | S-I | **0.000** | **0.750±0.049(0.655-0.845)** | **0.001** | **0.776±0.059(0.660-0.893)** | 0.193 | 0.625±0.103(0.423-0.827) |
|  | S-I (3D) | **0.000** | **0.270±0.050(0.172-0.369)** | **0.008** | **0.271±0.068(0.137-0.405)** | 0.103 | 0.344±0.104(0.140-0.548) |
|  | A-P | **0.006** | **0.657±0.054(0.552-0.762)** | 0.411 | 0.571±0.081(0.411-0.731) | 0.023 | 0.719±0.095(0.532-0.905) |
|  | A-P (3D) | **0.001** | **0.306±0.052(0.204-0.408)** | 0.458 | 0.436±0.081(0.277-0.595) | **0.012** | **0.258±0.092(0.078-0.439)** |
|  | L-R | 0.489 | 0.461±0.057(0.349-0.573) | 0.208 | 0.392±0.077(0.242-0.542) | 0.193 | 0.375±0.095(0.190-0.560) |
|  | R-L (3D) | 0.281 | 0.439±0.058(0.326-0.552) | 0.073 | 0.346±0.074(0.202-0.490) | 0.103 | 0.344±0.090(0.167-0.521) |
|  | Volume | **0.003** | **0.329±0.053(0.226-0.432)** | 0.063 | 0.340±0.081(0.181-0.500) | 0.090 | 0.338±0.086(0.168-0.507) |
| Left maxillary | S-I | **0.000** | **0.722±0.049(0.625-0.819)** | **0.003** | **0.754±0.063(0.631-0.878)** | 0.318 | 0.596±0.094(0.412-0.779) |
|  | S-I (3D) | **0.001** | **0.314±0.051(0.214-0.415)** | **0.009** | **0.274±0.070(0.137-0.410)** | 0.152 | 0.363±0.094(0.178-0.547) |
|  | A-P | **0.001** | **0.685±0.052(0.584-0.786)** | 0.098 | 0.642±0.073(0.499-0.786) | 0.019 | 0.725±0.088(0.553-0.897) |
|  | A-P (3D) | **0.002** | **0.320±0.052(0.219-0.422)** | 0.266 | 0.404±0.076(0.255-0.553) | **0.016** | **0.269±0.091(0.090-0.447)** |
|  | L-R | 0.306 | 0.442±0.057(0.331-0.553) | 0.197 | 0.389±0.078(0.235-0.543) | 0.103 | 0.344±0.093(0.161-0.526) |
|  | L-R (3D) | 0.063 | 0.395±0.056(0.284-0.506) | 0.197 | 0.389±0.078(0.236-0.541) | 0.077 | 0.330±0.087(0.161-0.500) |
|  | Volume | **0.001** | **0.318±0.053(0.215-0.421)** | **0.025** | **0.307±0.075(0.161-0.453)** | 0.193 | 0.375±0.093(0.193-0.557) |
| Sphenoid | S-I | **0.005** | **0.660±0.054(0.555-0.766)** | 0.175 | 0.617±0.081(0.458-0.775) | 0.501 | 0.565±0.099(0.370-0.759) |
|  | S-I (3D) | **0.001** | **0.317±0.053(0.213-0.422)** | 0.180 | 0.385±0.081(0.226-0.543) | 0.339 | 0.408±0.101(0.210-0.606) |
|  | A-P | 0.092 | 0.595±0.056(0.487-0.704) | 0.448 | 0.565±0.084(0.402-0.729) | 0.099 | 0.658±0.091(0.479-0.837) |
|  | A-P (3D) | **0.019** | **0.367±0.054(0.260-0.473)** | 0.529 | 0.446±0.080(0.288-0.603) | 0.051 | 0.313±0.090(0.137-0.488) |
|  | L-R | 0.308 | 0.442±0.057(0.331-0.553) | 0.084 | 0.351±0.077(0.201-0.501) | 0.200 | 0.377±0.103(0.175-0.580) |
|  | L-R (3D) | 0.153 | 0.419±0.057(0.308-0.530) | **0.045** | **0.328±0.072(0.186-0.470)** | 0.216 | 0.381±0.102(0.181-0.582) |
|  | Volume | **0.037** | **0.382±0.055(0.274-0.490)** | 0.156 | 0.378±0.082(0.218-0.538) | 0.250 | 0.390±0.088(0.217-0.562) |

S-I: superior and inferior planes distance; A-P anterior and posterior planes distance; L-R-: left and right planes distance; S-L-: superior and left planes distance; S-R-: superior and right planes distance; A-L-: anterior and left planes distance; A-R-: anterior and right planes distance; S-A: superior and anterior planes distance; S-I (3D): superior and inferior points distance; A-P (3D) anterior and posterior points distance; L-R (3D)-: left and right points distance; S-L (3D)-: superior and left points distance; S-R (3D)-: superior and right points distance; A-L (3D)-: anterior and left points distance; A-R (3D)-: anterior and right points distance; S-A (3D): superior and anterior points distance.

**Supplementary material 4**

Table. Final selected hyperparameters for the best-performing machine learning models.

| Method | Hyperparameter | Selected value |
| --- | --- | --- |
| RF | n_estimators_range | 100 |
|  | criterions | entropy |
|  | max_depth_range | 10 |
| LR | C | 10 |
|  | Solver | liblinear |
|  | max_iter | 200 |
|  | penalty | l2 |
| SVM | C | 1 |
|  | penalty | l2 |
|  | loss | squared_hinge |
|  | dual | false |
|  | max_iter | 500 |
| KNN | n_neighbors_range | 5 |
|  | p_range | 2 |
|  | metrics_list | manhattan |

RF; Random Forest tree, LR; Logistic regression, SVM; Support vector machine, KNN; K-Nearest Neighbors.
